#  Intestinal Infarction in COVID-19 Pandemic: A Case Series

**DOI:** 10.30699/IJP.2021.525280.2600

**Published:** 2021-11-07

**Authors:** Maryam Sarkardeh, Amin Dalili, Naser Tayyebi Meibodi, Mostafa Izanlu, Seyed Javad Davari-Sani, Saeed Moghaddamzade, Mehdi Jamalinik, Seyed Javad Hosseini, Javad Koushki, Ali Abedi

**Affiliations:** 1 Surgical Oncology Research Center, Faculty of Medicine, Mashhad University of Medical Sciences, Mashhad, Iran; 2 Department of Surgery, Imam Reza Hospital, Mashhad University of Medical Sciences, Mashhad, Iran; 3 Department of Pathology, Imam Reza Hospital, Mashhad University of Medical Sciences, Mashhad, Iran; 4 Student Research Committee, Sabzevar University of Medical Sciences, Sabzevar, Iran; 5 Department of Nursing, Islamic Azad University, Tabas Branch, Tabas, Iran; 6 Department of Nursing, Esfarayen Faculty of Medical Sciences, Esfarayen, Iran

**Keywords:** Abdominal CT Scan, COVID-19, Mesenteric Ischemia, Thrombosis

## Abstract

Novel coronavirus disease 2019 (COVID-19) as a potential health risk factor continues to spread worldwide. Although common symptoms include headache and respiratory symptoms, some studies have suggested that COVID-19 may cause coagulation disorders and thrombolytic events, disrupt blood flow to the visceral organs, and cause some complications such as mesenteric ischemia. The authors reported four cases of acute mesenteric ischemia associated with COVID-19 confirmed in patients hospitalized in Imam Reza Hospital (a COVID-19 referral center in Mashhad University of Medical Sciences, Mashhad, Iran). The authors described the pathological findings that may be associated with this infection. The authors collected clinical data, imaging, microscopic, and operative findings of four patients with severe COVID-19 infection and evidence of intestinal necrosis. These four cases of severe COVID-19 pneumonia simultaneously showed intestinal necrosis during the infection process, indicating a relationship between coronavirus and mesenteric vascular events. Physicians should be aware of thrombosis symptoms in the digestive system in patients with severe COVID-19.

## Introduction

Novel coronavirus disease 2019 (COVID-19) is now a worldwide pandemic caused by severe acute respiratory syndrome coronavirus 2 (SARS-CoV-2) (1). New evidence suggests that venous and arterial thromboembolic disease due to hypoxia and hyperin-flammatory response to COVID-19 may lead to the extensive amount of inflammatory cytokines and activation of different coagulation pathways (2). Also, in patients with severe COVID-19, mild to moderate lower platelet count (thrombocytopenia), elevated D-dimer, increased fibrin destruction products, and prolonged prothrombin time have been reported (3). Numerous studies have suggested that there is a link between gastrointestinal symptoms that are seen in patients with COVID-19 and coagulation disorders such as excessive coagulation and intestinal necrosis (4). Intestinal necrosis is a late symptom of various disease processes, which eventually causes cell death as a result of reduced blood flow to the bowel. These severe and deadly conditions are often caused by acute vascular occlusion and, less commonly, bowel obstruction infection or inflammation (5). As a result, based on the findings, hypercoagulable state, blood stasis, and endothelial injury increase the risk of thrombosis in patients with COVID-19 (6).

## Case Report and Methods

In this case series, we presented four cases infected with COVID-19 confirmed by reverse transcriptase-polymerase chain reaction (RT-PCR); they were referred to Imam Reza Hospital Complex, Mashhad, Iran, with abdominal discomforts; the laboratory tests, imaging, pathology, and operating findings suggested intestinal necrosis.

Case 1

**Fig. 1:**
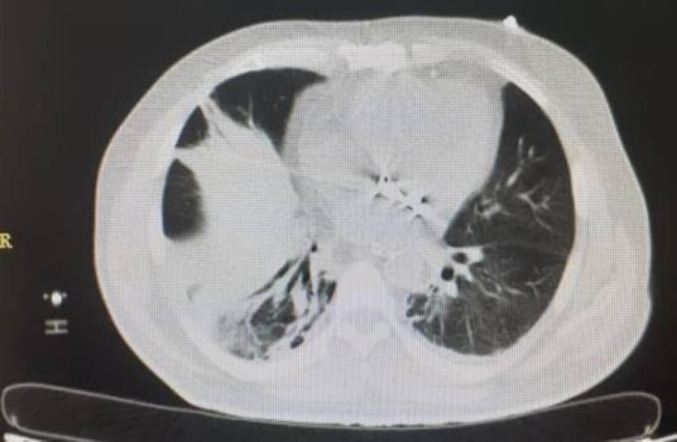
Lung involvement with COVID-19

**Fig. 2:**
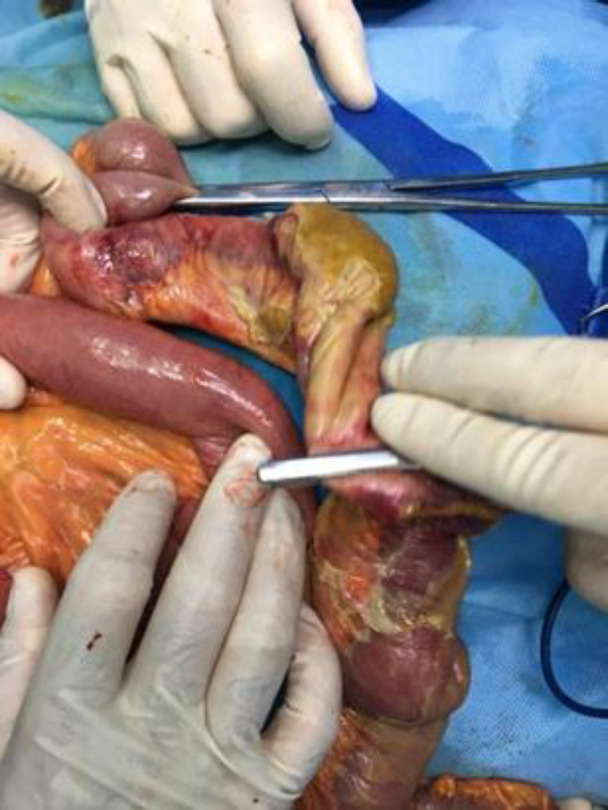
Intestinal ischemia and necrosis that it was resected

**Fig. 3:**
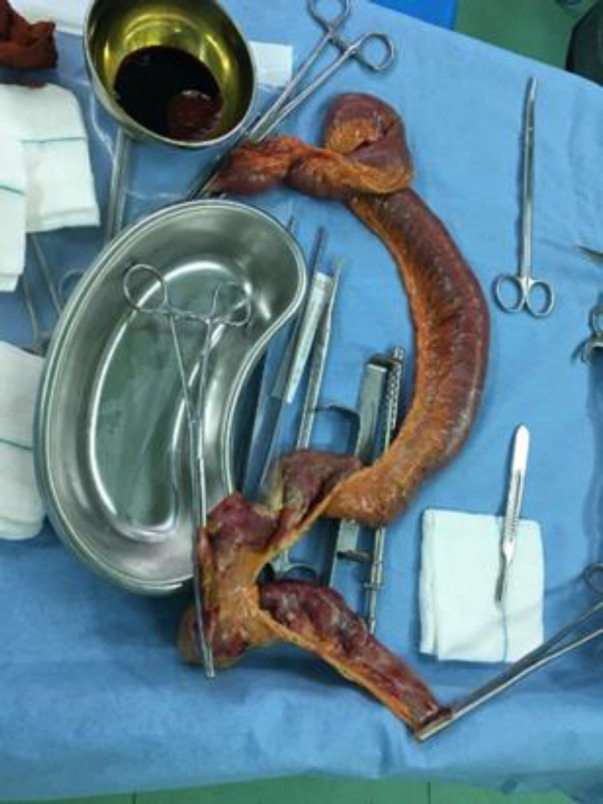
Intestinal ischemia and necrosis that it was resected

A 58-year-old man with COVID-19 confirmed by RT-PCR and chest computed tomography (CT) scan (Figure 1) was referred to the emergency department with a history of mitral valve replacement, hyper-tension, and complaint of generalized abdominal pain and dyspnea. Vital signs on admission time were as follows: blood pressure (BP) 140/70 mm Hg, pulse rate (PR) 120/min, temperature 37.3°C (oral), and oxygen saturation (SpO_2_) 92% in room air. He had a relative decrease in white blood cell (WBC) count. On abdominal examination, he had tenderness in the right lower quadrant (RLQ) without any guarding and rebound tenderness. He used warfarin due to cardiac problems; the international normalized ratio (INR) was 8 upon arrival, and no cardiac thrombosis was seen on his echocardiogram. After an abdominal CT scan due to abdominal pain, mesenteric ischemia was diagnosed. After correction of INR with transfusion of fresh frozen plasma (FFP), surgery was planned for him. During the surgery, 140 cm of the small intestine in the ileum was ischemic (Figures 2 and 3). After the operation, he suffered a drop in SpO_2_ and BP that led to his death.

**Case 2**

**Fig. 4:**
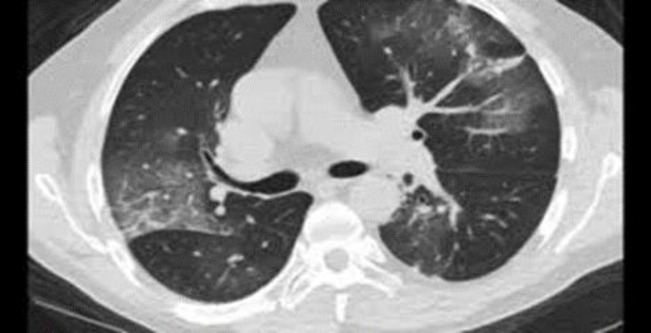
lung involvement with COVID-19

A 51-year-old man with confusion and a history of diabetes and end-stage renal disease (ESRD, who was on dialysis treatment for years) was referred to the emergency department. On arrival to the emergency department, he had a fever, respiration distress, low SpO_2_ level (92% with additional oxygen), and abdominal pain. High-resolution computed tomo-graphy (HRCT) was performed due to the patient’s dyspnea, which was in favor of COVID-19 (Figure 4), and two days after the PCR test, it was reported positive. Blood tests also showed high levels of amylase, INR, Partial Thromboplastin Time (PTT), lactate dehydrogenase (LDH), and thrombocytopenia (75,000/μL). In the primary complete blood cell count (CBC) test, the hemoglobin level was 10.9 mg/dL. In the lower cuts of HRCT, free air (pneumoperitoneum) was seen. The patient underwent an emergency laparo-tomy; during the operation, necrosis and perforation in three parts of the ileum with a length of 40 cm at a distance of 70 cm from the ileocecal valve were seen; after the operation, the patient’s condition became unstable that led to his death. 

**Case 3**

**Fig. 5:**
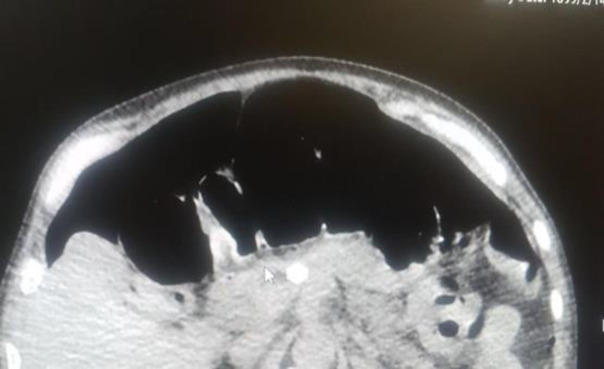
Pneumoperitoneum in CT scan

**Fig. 6:**
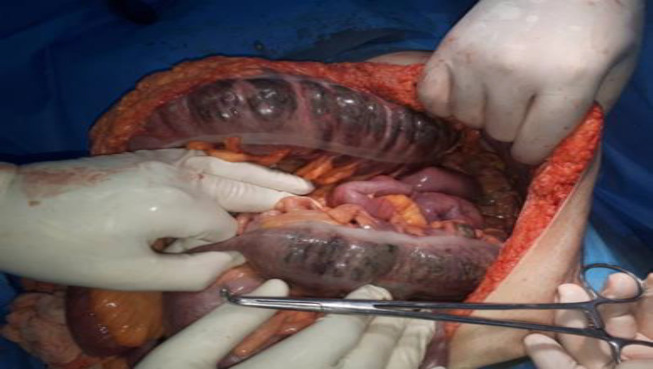
intestinal ischemia and necrosis

A 60-year-old man was admitted to the intensive care unit (ICU) due to COVID-19 with no past medical history, and infection was confirmed by the COVID-19 PCR test. Four days after admission, the patient complained of abdominal pain and inability to expel gas and excrete feces. The patient’s consciousness decreased the day before the surgical consultation. The surgeon reported tenderness and rebound tenderness during the physical examination. Free air was confirmed by an abdominal CT scan (Figure 5). Blood tests also determined a mild decrease in platelet (130,000/μL) and an increase in Prothrombin time (PT), Partial thromboplastin time (PTT), international normalized ratio (INR), lactate dehydrogenase (LDH)

bill direct, Aspartate aminotransferase (AST), and Alanine aminotransferase (ALT). Due to signs of peritonitis and free air in imaging, the patient underwent emergency surgery. In this regard, a midline laparotomy was performed, and macroscopic observations revealed that a 60-cm-long intestine near the ileocecal valve and entire colon was necrosis (Figure 6). Unfortunately, two days after surgery, the patient died due to respiratory arrest and hypotension.

**Case 4**

**Fig. 7:**
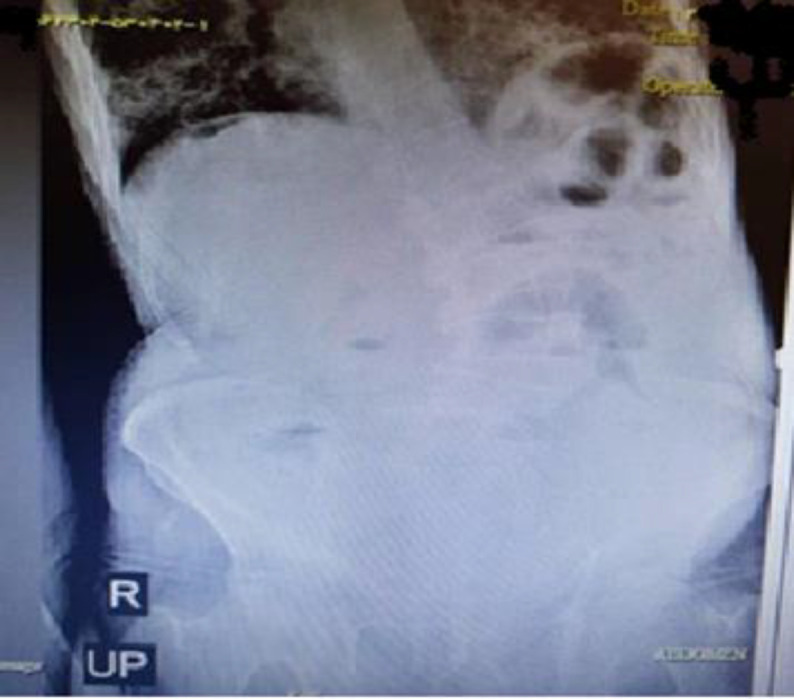
Abdominal X-ray, upright and supine

An 88-year-old man with a diagnosis of COVID-19 confirmed by PCR was referred to the emergency department due to respiratory distress from one week ago and melena (several times) from three days ago, abdominal pain, bloating, and inability to expel gas and excrete feces. During the abdominal examination, he had generalized tenderness, rebound tenderness, and abdominal distention. He had anemia and low hemoglobin and hematocrit, and the levels of urea and creatinine were high. Considering abdominal exami-nation and final assessment (Figure 7), a diagnosis of peritonitis was made. Accordingly, the patient was prepared for emergency surgery; during the operation, about 130 cm of the ileum was resected due to necrosis. Unfortunately, two days after surgery, the patient died due to respiratory arrest.

**Histopathological Findings**

**Fig. 8:**
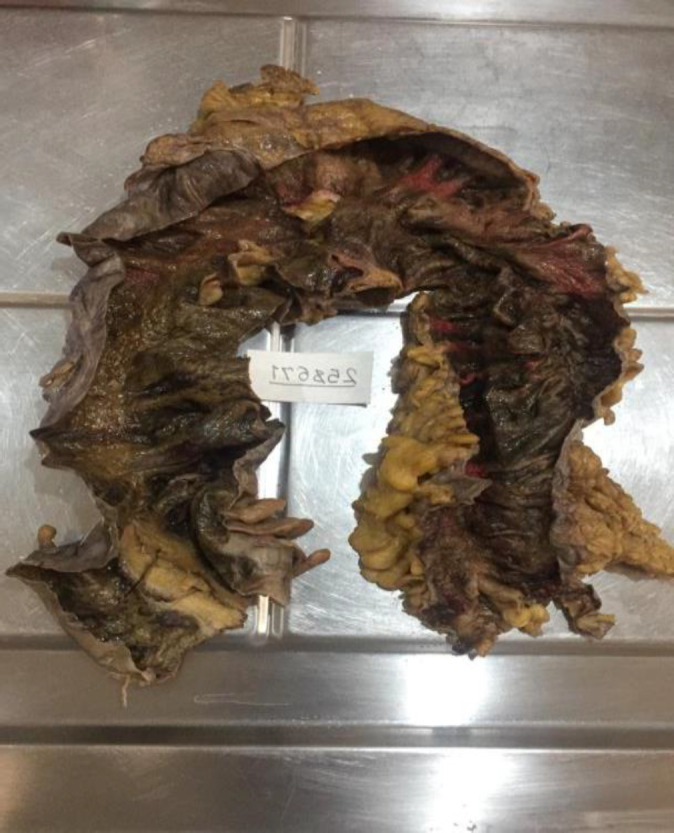
The outer surface was brown and congestive appearance, segmental thinning in areas of full-thickness infarction, luminal surface with multiple foci of mucosal ulceration

The macroscopic and microscopic findings were same in all cases. All samples in the gross description presented with segmental thinning in areas of full-thickness infarction, and the outer surface was brown and had a congestive appearance. A luminal surface with multiple foci of mucosal ulceration was reported (Figure 8).

**Fig. 9:**
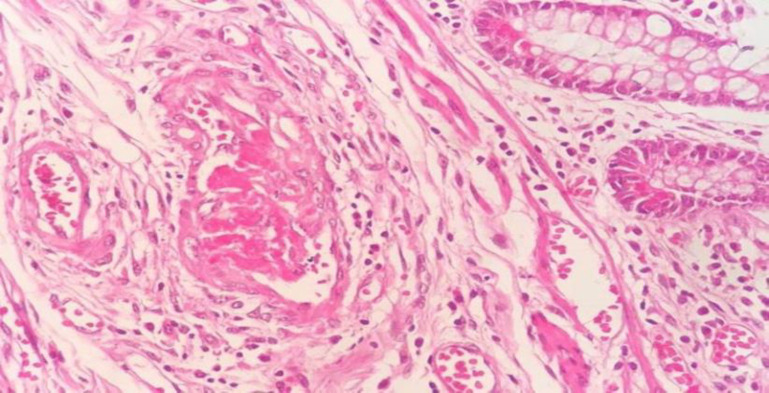
Intraluminal thrombus with vascular wall and perivascular neutrophilic infiltration

**Fig. 10:**
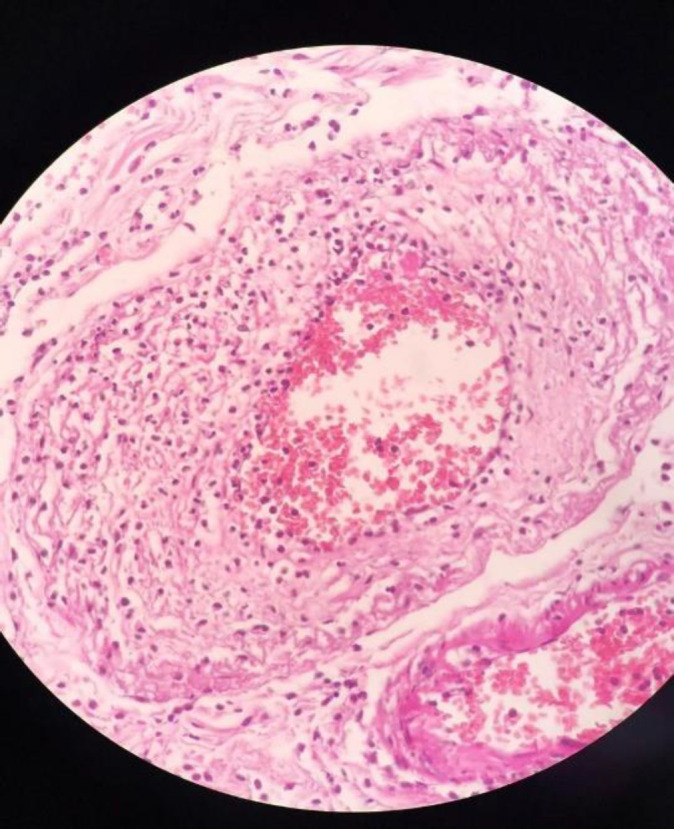
vascular wall and perivascular neutrophilic infiltration

**Fig. 11:**
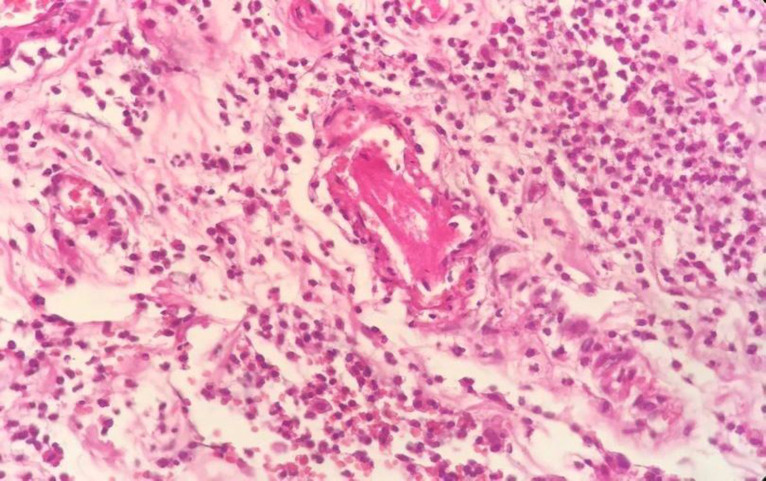
**Intraluminal thrombus and perivascular prominent** **neutrophilic infiltration**

In the histopathologic evaluation, microscopic findings showed identical results, perforation in three cases, multiple ulcerations, coagulative and liquefactive transmural necrosis, intraluminal thrombus with vascular wall, and perivascular prominent neutrophilic infiltration (Figures 9–11)**.**

## Discussion

Recently published COVID-19 studies have shown that coagulation abnormalities occur in patients with COVID-19, including hypercoagulation status, thromboembolic disease, and ischemia (7, 8).

The pathophysiology of hypercoagulability in COVID-19 is vascular endothelial dysfunction, causing microcirculatory changes in SARS-CoV-2 (7, 8). The virus adheres to a receptor by the angiotensin-converting enzyme 2 (ACE2) receptor on endothelial cells (9). Then, viral replication causes inflammatory cell infiltration, endothelial cell apoptosis, and microvascular prothrombotic effects (9). Polyphos-phates formed from viral antigens activate platelets, mast cells, and factor XII, and they activate other coagulation pathways as well (10).

Ignat *et al.* reported a mesenteric and portal vein thrombosis in patients with SARS-CoV-2 (11). Rossana* et al.* indicated abnormalities in medium and small size pulmonary vessels in COVID-19 patients due to perivascular inflammation; endothelial cells appeared large, massive liquefactive degeneration of endothelial cells, multiple intraluminal thrombi (12). Louis Maximilian Buja* et al.* collected pathological findings from 23 autopsies; they reported several cardiopulmonary events due to microthrombosis in small lung vessels and intravascular trapping of neutrophils and endothelial damages in heart and lung tissues (13).

Guidelines suggest that patients with COVID-19 infection should receive prophylactic doses with low-molecular-weight heparin (LMWH) unless there is a contraindication to anticoagulant therapy (13). The question now is whether thromboprophylaxis should be used in outpatients. Several studies have reported that for patients with thrombotic risk factors (such as venous thromboembolism [VTE], immobilization, and recent surgery), anticoagulant therapy is useful (14).

Consistent with several recent studies, these four COVID-19 cases showed intestinal necrosis during the infection process, which may indicate a relationship between coronavirus and mesenteric vascular events. However, further studies with more cases are needed to confirm this relationship. 

## Conclusion

These cases showed that prothrombotic consequences of COVID-19 are arterial and venous abdominal thrombosis, especially in severe cases. Therefore, in SARS-CoV-2, more attention should be paid to arterial and venous abdominal thrombosis, even in the absence of obvious signs of intravascular coagulation. In this paper, the cases had an underlying risk factor in addition to COVID-19, and the role of other risk factors cannot be ignored; accordingly, further studies with a larger sample size are needed to investigate the relationship of other risk factors with COVID-19.

## Conflict of Interest

The authors declared no conflicts of interest.
